# Supplementary material for: Genome-wide identification of hypoxia-induced enhancer regions
Source: PeerJ. 2015 Dec 21;3:e1527. doi: 10.7717/peerj.1527 (PMC4690393; doi:10.7717/peerj.1527)
Supplement: File S1 [file peerj-03-1527-s001.docx]

**All oligonucleotides are listed 5’ to 3’**

*= Phosphorothioated

Phos = Phosphorylated

**Genomic-Adapter-1:** ACACTCTTTCCCTACACGACGCTCTTCCGATC*T

**Genomic-Adapter-2:** Phos-GATCGGAAGAGCGAGCTCGCCCGGGGATCGAGCGCAGCGG

**Illumina P5:** AATGATACGGCGACCACCGAGATCTACACTCTTTCCCTACACGACGCTCTTCCGATCT

**Genomic-R:** Phos-GCGGCACGTCTGCTCGGCTCGAACATTCATTCACAACTGATGCTCTCAGCCACCCCGCGCCCTTTTATACCGCTGCGCTCGATCCCCGGG

**GFP-F:** ATGGTGAGCAAGGGCGAGGAGCTGTTCACC

**SV40-R:** TTAAGATACATTGATGAGTTTGGACAAACCACAACTAGAATGC

**Marker-1-F :** GATCGGAAGAGCGGTTCAGCAGGAATGCCGAGACCGATCTCGTATGCCGTCTTCTGCTTGATGGTGAGCAAGGGCGAGGAGCTGTTCACC

**Marker-2-F:** CTCCACAGGTgtcGAATAAGCCAACTTTGAATCACAAGACGCATACCAAACNNNNNNNNNNNNNNNNNNNNAGATCGGAAGAGCGGTTCAGCAGGAATGC

**Marker-3-F:** Phos- GCCTTCGTTAATATCCTTTGGCAGGTAAGTATCAAGGTTACAAGACAGGTTTAAGGAGACCAATAGAAACTGGGCTTGTCGAGACAGAGAAGACTCTTGCGTTTCTGATAGGCACCTATTGGTCTTACTGACATCCACTTTGCCTTTCTCTCCACAGGTGTCGAATAAGCCAACTTTGAATCACAAGACGCATACC

**Illumina-P7:** CAAGCAGAAGACGGCATACGAGATCGGTCTCGGCATTCCTGCTGAACCGCTCTT CCGATCT

**RNA-BC-1:** AATGATACGGCGACCACCGAGATCTACACTCTTTCCCTACACGACGCTCTTCCGATCTTCGGGTAGCCAACTTTGAATCACAAGACGCATACCAAAC

**RNA-BC-2:** AATGATACGGCGACCACCGAGATCTACACTCTTTCCCTACACGACGCTCTTCCGATCTATCCATAGCCAACTTTGAATCACAAGACGCATACCAAAC

**RNA-BC-3:** AATGATACGGCGACCACCGAGATCTACACTCTTTCCCTACACGACGCTCTTCCGATCTCCTGTGAGCCAACTTTGAATCACAAGACGCATACCAAAC

**RNA-BC-4:** AATGATACGGCGACCACCGAGATCTACACTCTTTCCCTACACGACGCTCTTCCGATCTGTACCGAGCCAACTTTGAATCACAAGACGCATACCAAAC

**RNA-BC-5:** AATGATACGGCGACCACCGAGATCTACACTCTTTCCCTACACGACGCTCTTCCGATCTGCACACAGCCAACTTTGAATCACAAGACGCATACCAAAC

**RNA-BC-6:** AATGATACGGCGACCACCGAGATCTACACTCTTTCCCTACACGACGCTCTTCCGATCTAGCATAAGCCAACTTTGAATCACAAGACGCATACCAAAC
