# Supplementary material for: Genome-wide identification of hypoxia-induced enhancer regions
Source: PeerJ. 2015 Dec 21;3:e1527. doi: 10.7717/peerj.1527 (PMC4690393; doi:10.7717/peerj.1527)
Supplement: File S3 [file peerj-03-1527-s003.docx]

>2L:2887100..2887600

TGCAAATGCTATAAGTAGGAGGGGGAGAGAGAGTCACACGCACACACACCTCAAGCAATTCTGATAAGAACAATTGTTATTTGTTTGAAAGCCGACATTCCAATAGTCCACGTCCATTCGCCCAAACGTTGAAAATAATCGACAAACTTAAATACTATTGTGTTTGTTCGCGAACCCAGAGATGCTTTGCCAACTGATGAAATAAATATGGCCAATGCCGTCAGAATAAAGCGAAAATACCATTGTGGTTGGAATTCTCTTAATGTTAACATTGATTACTCCATTTTCTACTTTCTAAACAATGTATTGTATTCAATATTTATATTGATAATATCGAAATAGATAATTGCAGTATTTAATTGTTTTTGTACTTCGAATTAGAAATATGACTTTAATCACGTACGCAACAACCCATGCTTTCTTCCTAAGAAATGACAAAATAATAGTCTCCACGAAATAGGAGAACTCCATACTCAAAATATAAGCTTCAGAAGTTTATA

>2L:5986900..5987500

CAATCGGTTCGAATTCATACCCTAAACCGCACCCTCACGCACACATGGGCATTAACACTTTTCTCTGGGCAATAGGGCGTAGTCACACAGTAAACAGCGAAAACATCATCCCATTCCGCATTTATCCATTTAAAAGCCGGCTTGTGTTGTTCAACACTGATTGCTCTCCATTGGGTTTCATGTGAATTTTCACCCCGCAAACTTGTGAATCAGTTTTCATGTTGTTCCCTTTTGCCGGGCATCGAGTGCGTCTTAATTGCGCCGATTAGATAAGGTTGTAGTTATATGTGTATCCGAATAGGCGCTCAATTGGTCACGTAGCTCAGTGATTTGCCACTCGACTTCTCGGCTGCAAGTCGCGTTCAGTTGAGTCACTGCTTCGCCTTCCGCAAAAAGTTCCCGAAATATTATTCAAACTAATTTTATGAGTGTTGTTTATGTTTACAGTTACAGTCATTCACTATGTTTCTAGTCGTAGATAGTGTTTATCTTATCGCCCAGGTGCACCATTGGGCGGATCATTGATTGTCGTTCAACAACGTCCCAATCCCAAATGTTCGCTTCTCAGTCCCAACTAAGTCTAAAATAAGTCATGTACGT

>2L:8001300..8001800

CAACTTTTCCCACGCAAAAATATAATTATTTGAAAAAGAGAAACCTTAAATAAAAATATTTTATCTATGCAACAATGAATAACCCAATTTTTGTGAGCTGATGCAAGAATAACTAATTGTAAGTAATCTTTAATGTTTGAAAATGATAACCAGTCGAACTTCCATACCAACATTCATATCTTTTTCTCGCCCGTGTGAACGTGCAACAAAAGTCTCGTGCGCCACATTGGAGTACTCGCAGATAGTCATGTATGCTCCGCGAAGCCAAAAAATGTTGTACGTGAGCACAGCAACGAACGCAGCAGATACAAAACCACCTCCAATAGCCAGTATGTATGTACATACATATGTACATCCAGTTCAGTTTGAATTGGTATTGTGCCTCTTCACGAGATTCGCTCACCCAGTGATTTATTTGGTCTTTGACAGCATGCACAGTGAGCCATTCGAGCGGCCACAAATGAATCAATCTAATTAAGCGAGTTAGCGCTGATAAGGAA

>2L:20082900..20083500

TGCTTCCTTTGCTCCCGCTTGCGCATGTAGTTGATGAGCGGCCGCTTCTTGTGCTCCTTGCAGAATGGGCAGTCCTTGCGCATCTGCTTCTCGGACTTTATCTGCTTGCACTTTTCTGCAATGGGAGCAATGTGATAGGTCACCGGTTAGTGAAGGTCGGCACTGAATGTTCACCCTCGTCTGTTGTTTCCCGCATTCGCTTCCACTCTGCCTTTTAGCCTACGTGCCCTTCTGGGCCGTCGAACAGCTGATTTGACTACGTGACGTCGCTGCGTCTACGTTCACGACGTCACGTCGCCACCGATGGCGCCCCTCCCCACGCTCGCACAATTTTTCCCGCTACTCGACGTGTGACCCTGTGACGGAGGCAGCTATATATTTAATATTATCATGCACTGGATTTTAGAAACCTATTTTAAAAAGTTGAATCTATCTAATCTTTGGTTTTAATGAAAACATTGTTGTCTTTCGACGACTGTAACACAACTACTACATTGAGCACAACTGTACTTACAGCTTGTGTGCTTTTGTGACCTGCCTCCGTTAATTAAACCATTGCTTTGATTGCTCAATTGACCGTGTTAAAAGCTAAACGGTTAT

>2R:12896000..12896500

CGTGCTCCAGTTTCCTCGCGATCTCCTGGTCGTCTTCGATCCTAAACGAGTCCGAGGGTTAGCTTCGTATATTTTAATGATGGGAAAAGTGCTTCACTTACGCTATCTCTTGGTTGTGCAAGTGCTGAACCAACTGGTCGCGCTTGTCGACAATCGTCATCAGTTCCTCGAGCAGCAGTCGCTCCTTTTCACGCTGAACTTCCGTTTTGCGCCAATCTTCCACAGATTGCGCCGCCCGCAGCTCTTGATTAAGCATTGTATATTTTCGCTCCAAATCTTTTTCTTGCTCGCTGAAGGGTGTAGATAATAATAAGAAACAAAGCCGATAGTAGTTCTAAGTGATAACTTACAGTATATTTAGCTGCATCTGTCGCCGGAGGAGTGCGTTCTTCTTGTTGACCAGCGTAAACCATTGCGATAGCAGTTGCTCCTCTGTCTCCTCGGTCTCTGTGGAATGTTTAAGTGTCTGATTATAAAAAGGCAATGATGGGGGTTTTCTT

>3L:3892900..3893100

GCTGTCGCCGCAGCTGGGAAATGCTGATGGCGCCCGTATCGCCCAAGAACCTCAGGACCACGGCGCTCTTGACGAGCATCTACCAGTTGGTAAGTTCAAACATACATGTATATATAGTAGCGGATTATATAAGAGACTGGAGAATGGATATCTCAGGCGGGGCGCAGCGCACTGGCACGCAGGGAGATCCGGGAATCCCC

>3L:6256700..6257200

TATCCCTTTAATCTTAAAAAGGACCAGCTTTAAGAACACTGTCTGTTTAATTGGTTGCCTTTAAAAACATATACTCATTGGATTCCGTGTATAATGCTTATAAGCATTTTATTAAGAGTGTATGGCATTTCACACCTATTGAGCTGGATTTAAGCAGACTTTCGCTAATTGTTTCAAATACGTCATCCCCTGGTCTTCTGTACTTGCATTGTGGTTAGACAATTCCATAGAGAGTCCTCAAGAGATCTGTAGAGAAAAATAGAAGCAGAGGGAGATCCCCAGAGAGAAGTTGCTCCACCAGTTTGCCGGCGTTCGTGACTGACAACACATTTTACAGACTCAACATTTATTCGCCGGTGATTCACTGCACTCTCTCGATTTTCGCAAGCATTTCGACTTAATACGTCATCATTGCCGTCGGCTAGCTTGAGTTGTCAAAGCGGGGAAGGGGGTGGTGATGAGCGGTTTATTGCTCTCTCAATGTCACTGCAATTTAACGC

>3L:7797800..7798600

AAGGCATCTGAAAAACACACAGAGAACAAAGAGAATATGAGTTGGATTTTGATTTCGATTCCGGGAGAAATATATGTGCATATATATGTATGTGAGAGAGATAAAGAGAGAATGTGAAGTAAGCGGAACAAGAAGAAGATAGGGGTTTTACGTAACGCTCGAAAAAAAAAAGAATAAATAGCAGCCAAAGAAATGAAATGACTGATTCTCAGAGGCAGCTTTTGATTCTGTTTTATAATTCCCAGCCCACACACACACACACACAGACACACACAATCAAATCAAAGAGAGAACCCTCTCTCTCGCGCTCTTGGATTACTGGGTGGCCCAGGAAGAAGAGGAAGAAGAACTACAGTATCTATTACTTAACCACTGGAGAACGCCACAGATATCACGCACTCGAAATGCATTTCCCACATCACCAGGCCCAAAAAGCGATGACGTGACGTTCAGCACCTTAGCACAGAGCTAATTATTAAGGCACAGTTCTTATATAAACTGTCGAGTTCAATGAACTTTCGCAAAGTGCCCCCTGGAAGCCGTAGAAAGCCAAACTAAATCCATTAAATCGCCGCAAGGTAGGGCATAGAATAGAATCGCAACCAGGCGGCGGCACATGCTATTACATAATATTATGAAAATTAACACTGAATCACATAGAGCGAACTGACGCGGACGCTGGCTAAAGTAACGAAATCAAAATCATCGGCACACAGATACTTTTTATACCAATACTTGCGCACGTCTACCTTCTCTTAGCCGTTCTCTCTTACGTTCTCTTTACGAGAACCATTTCAGCG

>3L:8685300..8685800

ATTTTTTTTAATCTCAAACAGGTTTTACAATATCTTTAACCAAGTAAATAATAATATATTTTAAATCAATATAATTGATTTACATTTATTAAGACCGTAGATTCTTATTATTTACTTGTCTATACCCAGTACTTTAAAAACTTTGAATATATACTGTAAACTTATTGATTGTATATTTACTTATTTTCAACACATGGTAAAATGTATTTTCGTATCTTAAAGCAAGAACCTAACAGTTTTTTTCAGTGCACAGTTCGAAGGCGTCTACGTGCGCTGCAATGCGAAGCACGAGCCCCGTCGTTAATTTCACCATTAGCCTCCTCGCACAGTGAACACTCTGATAAGACGCCGGCATGCCGTGCCACACATGCACACACATAAATATATAAAGTAATATATGACTGATTGTCTGCCGAGGCCCAAGACGTCGGTCGTCGTAAACAGCTTATCAGCCGGGCAATGTGGTTACCCGTCGAAAGCGCTGGAACGGCAACCAGATC

>3L:9385200..9385800

TGAAATATCTTTTGTATATCAAGAGTAACAACTTTGCACCGGATGTATATCAATATTATATATTGTATATTATATATTGTATACACAGAAGTTGAACTCTTATGTTCCTGTGATGATGAAGTACATTATCTGAAATCGCCATTTCTAAAATATTTTCAAAAAATGATTCGTATTACTGTTAAGCAATTATAAGCACAGATGAAACACACATTTTTCCTGATAATTTTATAACTTTAAATGTATCAGATTTTTCGTTTGAAAAGAGCATCAACCCAATGACTAATTTCCACTTATTTCGCCACAGTGTGGGATGGCTTCCCATCATCAAAACACCCTTCGCCGCTGCCCCCTGCCAATCTGCACTATCCCAAGTACTTTTCTTCCCTGTTCGTCCGTCTGTATCTGTAGTTTTTAGAGTTCAGTTCAGGCAGAGCTCACACAGTTCATTTACGAAAAAAACATCGACGTAATGCTACTATCAGAAACGCACCTCACAAACAAAAACAATTTTCATATACCAGGATACTTGTTCTATGGTACAAATCATCCAGATGGTAAAGCTCATGGAGGCACTGGAATACTCATCAGAAATCGCATAAA

>3L:9448800..9448900

TATATATTCGCCAGACTTCCAAAGTGGACAAACGTACGTGTTTTCGTCGGTATTGCTGCACTGAACTGGCCTTTCCCTCCACCTCTTTTTCCACTTTTTT

>3L:11234100..11234900

AATGTTTCGTATCACTTTTCAAAAAACGCAAACATGCAAGTCAGATAGAGATTAGCATTTAATGCAATAATAATTTCTATTGCACCTTTCCTTATTTTTTTTTTTTTTTTTTGTTCCAACCTACGTAGCTGATGAAATCGTCAAGGAAATATATAATCTCTTTTAATTGAACATAGAATTAAATGAAAACGTTATTTTCAGAACGAGTTTTCGCATGGAAACAGAATGTTGAGTCAGCGTCAGATGTTCAATAAATGGCAATAATATATGTAGGTTCCAAAGTTTTGGGCTATACGTAAGTGGATTTCAACCAGGGAGTATTACTTCGACTCACCGCTTTTTTTTTTTGAGGGACTTGGCTGACGTGTTTTTCAGCACGAAGCCATGATGAAATTATTAAAGCCATTTTAGCAGAGAAACCGCACTGACTAATGCCAAATGCGAAATCGGTTGCTATGGGCCCCATTTAACTAGGAAACTTAACTAGGACTGATGCAATCCACTCGCAATCTGAAAAAAAAGAGTTCGGTGGCTGGCACTTTGGGTAACCAAGAAGATAATGCCACATATGATTTAGCTAATTTCTTTAAATTGTTTTAAATTTATTTAAAATCTTCAATATATGCTTATTTCCGCCGGAAATTGCAGTGATTCATAAACAGACAGCATAAAAAATGACACATTTCATGTCATGAGTTTTTCACGATACCATTCTTTTCTTCTTCAGCTTTCTTCAAACTTTATGCATATATGAAATTTGTATTGTTTTACCTGAATAAGAGTAAAATTTTTAATAAATA

>3L:11522800..11523300

TCTTAGTCGATTACCGATTCGTGCGACAACTTTATTGCACCTCCGATCGAATGGGAACTACTGGCAAACAAACCATTAACCCACACACTCAATTTACACACTGGGGCCAATCGAAGTGACGTGTTGTGTGGCGCGATAAGTCGACTTCTTTCGGTTTCTTGCAATTTCACAAGAAGTTCCTCGGAAACGTCACATACAAGTCGTATTAAATGTGTGTTTTATGGGGTTTCCCGTATAATATCTTCTATTTTTCTTTTATCACATAAACACCTCGCTTCTATATCGCAGAATAGGCCGCAAATATATTCGCACGTTTGGATTTTATATCGCATTTCGTGTGCCATCGCTACGAGTCGAGCGAAAACCTAAAACTAAAAGCCACAAAAAATCGAAATGGATGGATGGTATGGCCAAAATGGGTGCATAAAAAATTGGTGATAAATAAAATTGAAATTCCAACTCATCATTTTCGTATTGGCACGAAAGAGTGCCCAAAATGT

>3R:2902300..2902600

GCGCAATTACAGTTAACGGCAAAGTTAAAAAGTTGAGGGCAGCGCAGCAAGAAAAATAGATGAGTTTCTATATACATACATACATACATATGTGTGTAGAGAAACCGTCGATAAGGTGGCAGAAGGGCAGAACAAATGGGTCTCTCGTTTTCACTCCACTTCGCCAAGATTTGCCAGTGGTGGGGTGATGTCGGATGACGGGCGTCATTGTGCCGGGCGTCACATCCGCAAGTGGCGTGACTGAATTTGTAGTCTAGGGTACTCGTATTTCCGACCGCATCTGATTCACTTTTCATTGTT

>3R:4181100..4181600

TTTATTTTTAGACACCCACTGCAACTGCACTGCATCCGCTGAAAGTTTCCACATTCCACGCCGATTTCCATATGCCTCAGCATGTTGCTCATACGCCCGAGTCACCGCTTGAATGTGGATGGGGGGCTTGCCTTGGCGAATTTTTGGTAAACAACAGCATAAAATCATGCGTAATAAACTTACAGCTACGCGTTGCTACGTGCCGAAAGTCTCGCAGTTCCCCGGCTTCCAGGTTCCACCACCTTTTTCCAAGCGTGTGACAGTGAATACTGACAAAAAATGTATTTGTATCTAAAAAATGGAAGCCAAACTGTGTCAGAGCAAACGTTCAAAAGAACTAAATTCTGGATGAGCGTTAAGAATATACGTTAAAATACACAAATTTATTTGTCAACAGCATAATTTAATAGTTAAGTAAGACATATGCTGTAATATGGGGTTACATTTTTTTCAGTGCTAGATTGTGGATGGTATTGGATCTCTTTCTTATGACGCTTGTT

>3R:6800900..6801600

TCAAATGAAAACATGATAATATATTGTAGTTTTGTTTTACCTTTCGAAGAAAAGGGCCATAAAAAAGTGAATTTAATAGGAATCGATTACATAGTTTGTCAGATTTAAAAAATACAAATTAAAGACTGAATCACAGTACATTTTTAAAGGTTTTATTTGTATGAAAGAATTGGGTTTTGGAGAAACCAAAAAAGAAAACCAATGGGTACATTACAGGAGCCCACAAATCGCGATATCAAATGACCTGAGGTGTACTAATTAAAACCATTTTTAAATTAAACAACGCTGAGGAATATCACGTTCGTACTTTAATCAAATGAAATGATTTAATTTGTTTTTTTATGTTGATCAAAGAAGTACATGCTTTATAGCACGTATTTCTCTTTGCCAAATCGTCCCTTCTCTTTCAAATGGAAGCGCTTTAAATCACACTGGCGAATATCACATTTCAAAGTAAAGAAGAATTGAAATCAAAGCTTTTGCTGTACAAAGCCAAATAATCCATCAAGTGGCATTAACTTGAAACAATAAATTTCACACACAGCTGCAGAGGTAAGCCCAAACCAACATAGGTCTCGAACAACTAGAGCTCTGGCACTTGAAGTGCTTGACAACTAATCAGGTTTCAAATGCCTAAGCACACACACTCGTACACTGCTCGAAAAAAGTGTAAGAGTATCGGGCAAGGGGAAATAAATCC

>3R:7781900..7782700

GGACGGCGTGGTGCGGTTGCCCTGGTCGTTGGCGATAATCTCCACCTTGCCATGTTGGTAGACACCCACGCAGGAGTAGGTGGTGCCCAGATCGATTCCAATAGCAGGCATTGTGTGTGAGTTCTTCTTCCTCGGTAACGACTTGTTGAAAGTATTCAGAGTTCTCTTCTTGTCTTCAATAATTACTTCTTGGTTGATTTCAGTAGTTGCAGTTTTTAGTTTAATTACTTGGTTGTTGGTTACTTTTAATTGATTCACTTTAACTTGCACTTTATTGCAGATTGTTTAGCTTGTTCAGCTGCGCTTGTTTGTTTGCTTAGCTTTCGCTTAGCGATGTGTTCACTTTACTTGTTTGAATAGAATTGACTCTCCGTCGACGAAGCTCCTCTATTTATACTCCGGCGCTCTTTTCGCGAACATTCGAGGCGCGCTCTCTCGAAGCAACGAGAACAGTGCGCCGTTTACTGTGTGACAGAGTGAGAGAGCAATAGTACAAAGAGGGAGAGTCACAAAACGAATAGAGAATAACGGCCAGAGAAATTTCTCGAGTTTTCTTTCTGCCAAACAAATGACCTACCGCAACAACCAGTTTGTTTTGGGATTCTAGAATATTCGCTTTATTTTGGAAATTTCTTTATAAATACGGCTGCTTAAGTTAATTATGTTAGAGATAATCGAAGGGTTTGTTACGCGGATGTTGTCCGCCAGAAAGGCCTATGGAACTTTGACAAGATATTCTTAAAAATGTATTTACATACTAACTTAAAAAAGCTATTTATTTATTAGATTAATACAGACAA

>3R:7783900..7784500

GAAAAATAAGCCATAGTCGGCACCATAAGCATAACCTAGCTCTGCGATTATCTCTAACATAATTAACTTAAGCAGCCGTATTTATAAAGAAATTTCCAAAATAAAGCGAATATTCTAGAATCCCAAAACAAACTGGTTGTTGCGGTAGGTCATTTGTTTGGCAGAAAGAAAACTCGAGAAATTTCTCTGGCCGTTATTCTCTATTCGTTTTGTGACTCTCCCTCTTTGTACTATTGCTCTCTCACTCTGTCACACAGTAAACGGCGCACTGTTCTCGTTGCTTCGAGAGAGCGCGCCTCGAATGTTCGCGAAAAGAGCGCCGGAGTATAAATAGAGGAGCTTCGTCGACGGAGAGTCAATTCTATTCAAACAAGCAAAGTGAACACATCGCTAAGCGAAAGCTAAGCAAACAAACAAGCGCAGCTGAACAAGCTAAACAATCTGCAATAAAGTGCAAGTTAAAGTGAATCAATTAAAAGTAACCAACAACCAAGTAATTAAACTAAAAACTGCAACTACTGAAATCAACCAAGAAGTAATTATTGAAGACAAGAAGAGAACTCTGAATACTTTCAACAAGTCGTTACCGAGGAAGAAGAA

>3R:8293200..8293900

CGATAATCTCAACCTTGCCATGCTGGTAGACACCCACGCAGGAGTAGGTGGTGCCCAGATCGATTCCAATAGCAGGCATTGTGTGTGAGTTCTTCTTTCTCGGTAACTTGTTGAAAGTATTCTCTTCTTGTATTCAATAATTACTTCTTGGCAGATTTCTGTAGTTGCAGTTGATTTACTTGGTTGCTGGTTACTTTTAATTGATTCACTTTAACTTGCACTTTACTGCAGATTGTTTAGCTTGTTCAGCTGCGCTTGTTTATTTGCTTAGCTTTCGCTTAGCGACGTGTTCACTTTGCTTGTTTAAATTGAATTGTCGCTCCGTAGACGAAGCGCCTCTATTTATACTGCTGCGCTCTTTTCGCGAACATTCGAGGCGCGCTCTCTCGAACCAACGAGAGCAGTATGTCGTTTACTGTGTGACAGAGTGAGAGAGCATTAGTGCAGAGAGGGAGAGACCCAAAAAGAAAAGAGAGAATAACGAATAACGGCCAGAGAAATTTCTCGAGTTTTCTTTCTGCCAAACAAATGACCTACCACAATAACCAGTTTGTTTTGGGATTCTAGAATATTCGCTTTATTTTGGAAATTTCTTTATAAATACGGCTGCTTAAAAACTTATGTTAAAGATAATCGAAGCGCTTTGTTATGCGGTTGTTGTCCGTCAGAAAGGCCTATGTAACATTGACAATATACTCTT

>3R:8303000..8303500

GCTCTCCACTAAACTCTAACGGTTGTCTGCCGCGCATCAGAAAAATAAAAAGAGGCGCGTGCCAAGCAGAAACACGAAATTTCGTTTCATTTAAATGATCTGCGACATATACATACATATTACATACATATTTTTAACACTCCTTCTGCGCTTGTTTATTTGCTTAGCTTTCGCTTAGCGACGTGTTCACTTTGCTTGTTTGAATTGAATTGTCGCTCCGTAGACGAAGCGCCTCTATTTATACTGCTGCGCTCTTTTCGCGAACATTCAAGGCGCGCTCTCTCGAACCAACGAGAGCGGTATGTCGTTTACTGTGTGACAGAGTGAGAGAGCATTAGTGCAGAGAGGGAGAGACCCAAAAAGAAAATAGAGAATAACGAATAACGGCCAGAGAAATTTCTCGAGTTTTCTTTCTGCCAAACAAATGACCTACCACAATAACCAGTTTGTTTTGGGATTCTAGAATATTCGCTTTATTTTGGAAATTTCTTTATAAATAC

>3R:8327800..8328500

CAGAAGAAAGTCCGTTAATCGTTGATTTCGTTAACTAAAAGTACAAAATAATCTTTAATCTTTAGAAACGCAGCAATGTTTTGGCGGCATACGCATAACAAAGCGCTTCGATTATCTTTATCATAAGTTATTTAAGCAGCCGTATTTATAAAGAAATTTCCAAAATAAAGCGAATATTCTAGAATCCCAAAACAAACTGGTTATTGTGGTAGGTCATTTGTTTGGCAGAAAGAAAACTCGAGAAATTTCTCTGGCCGTTATTCGTTATTGTCTCTTTTCTTTTTGGGTCTCTCCCTCTCTGCACTAATGCTCTCTCACTCTGTCACACAGTAAACGACATACTGCTCTCGTTGGTTCGAGAGAGCGCGCCTCGAATGTTCGCGAAAAGAGCGCCGGAGTATAAATAGAGGCGCTTCGTCTACGGAGCGACAATTCAATTCAAACAAGCAAAGTGAACACGTCGCTAAGCGAAAGCTAAGCAAATAAACAAGCGCAGCTGAACAAGCTAAACAATCTGCAGTAAAGTGCAAGTTAAAGTGAATCAATTATAAGTAACCAGCAACCAAGTAAATCAACTGCAACTACTGAAATCTGCCAAGAAGTAATTATTGAATACCAGAAGAGAACTCTGAATACTTTCAACAAGTTACCGAGAAAGAAGAACTCACACACAATGCCTGCTATTGGAATCGATCTGGGC

>3R:8331100..8331800

ATCGTTGATTTCGTTAACTAAAAGTACAAAATAATCTTTAATCTTTAGAAACGCAGCAATGTTTTGGCGGCATACGCATAACAAAGCGCTTCGATTATCTTTATCATAAGTTATTTAAGCAGCCGTATTTATAAAGAAATTTCCAAAATAAAGCGAATATTCTAGAATCCCAAAACAAACTGGTTATTGTGGTAGGTCATTTGTTTGGCAGAAAGAAAACTCGAGAAATTTCTCTGGCCGTTATTCGTTATTGTCTCTTTTCTTTTTGGGTCTCTCCCTCTCTGCACTAATGCTCTCTCACTCTGTCACACAGTAAACGACATACTGCTCTCGTTGGTTCGAGAGAGCGCGCCTCGAATGTTCGCGAAAAGAGCGCCGGAGTATAAATAGAGGCGCTTCGTCTACGGAGCGACAATTCAATTCAAACAAGCAAAGTGAACACGTCGCTAAGCGAAAGCTAAGCAAATAAACAAGCGCAGCTGAACAAGCTAAACAATCTGCAGTAAAGTGCAAGTTAAAGTGAATCAATTAAAAGTAACCAGCAACCAAGTAAATCAACTGCAACTACTGAAATCTGCCAAGAAGTAATTATTGAATACAAGAAGAGAACTCTGAATACTTTCAACAAGTTACCGAGAAAGAAGAACTCACACACAATGCCTGCTATTGGAATCGATCTGGGCACCACCTACTCCTGCGT

>3R:8334400..8335000

CTAAAAGTACAAAATAATCTTTAATCTTTAGAAACGCAGCAATGTTTTGGCGGCATACGCATAACAAAGCGCTTCGATTATCTTTATCATAAGTTATTTAAGCAGCCGTATTTATAAAGAAATTTCCAAAATAAAGCGAATATTCTAGAATCCCAAAACAAACTGGTTATTGTGGTAGGTCATTTGTTTGGCAGAAAGAAAACTCGAGAAATTTCTCTGGCCGTTATTCGTTATTGTCTCTTTTCTTTTTGGGTCTCTCCCTCTCTGCACTAATGCTCTCTCACTCTGTCACACAGTAAACGACATACTGCTCTCGTTGGTTCGAGAGAGCGCGCCTCGAATGTTCGCGAAAAGAGCGCCGGAGTATAAATAGAGGCGCTTCGTCTACGGAGCGACAATTCAATTCAAACAAGCAAAGTGAACACGTCGCTAAGCGAAAGCTAAGCAAATAAACAAGCGCAGCTGAACAAGCTAAACAATCTGCAGTAAAGTGCAAGTTAAAGTGAATCAATTATAAGTAACCAGCAACCAAGTAAATCAACTGCAACTACTGAAATCTGCCAAGAAGTAATTATTGAATACCAGAAGAGAACTCTGAAT

>3R:14892300..14892800

GATGTTGCCGGGGCAGGAAGCAACAACGGCAACAGCTGCAGCTACAATAGCAACAGCAACATCAGCAGCAAGCTCACGCATATTGCACAACTTTTAGTGGGAGGATGAGGTGGGAGCTGGCAATTTCTTTTGCCAGTCTGTTTTTTTTTTGTAACAACAGCAATTATTAGCTGTTAGTTGTAGGCAACATGGAAATTCGATATCAAATTGAAAAAACCCGAGGGGTAAGGAAAAGCGAATGTTGTTTTGGCGGAGGCGGGGACAATTAACGCATACTAAACTAAAAACCGCTATGCATCACGAACACGCGGAAATTGCCAGTGCACATCTCATGTATTTATCAGCTAAGCTCAAAGTAATAATAATAATAATAACAAACATTGCATAATCAATTAAATCTGACAAGGAGGCCTCACTTGCTATCAGCCTTCGAGATCACCTTCAGCAGTTCATTGCACGTTCGTTCGCTGGCTGGGTAGATGGGTTGGACGACTAACTGC

>3R:21433600..21434000

AGAACCCTAAGCGCTGAGTTGAAAGATGAACCAATTTAAAGATACACTGATGCACACTGTGTTGTTGTGTTGGTCGTTTTAAGAAAAGAAATTAGTAGCGTATATGAAGAGTATTTAATCTCAAAAAAATTAAATGTTTTACGTTGCCAGTGTTTGTGCAATACAAAATTATATAACAAAAAAATCGAGTCGAGAAGTTTTCGAATTGCCGTTTCTAAACTTGGTTGCCCTTCGCTTCTTATGCAGTATAAATATATTATTGAAAGTCAAATATTATATAAAGTATTCATAGATAAAGTATTTGATTAGACCGTAGTACATACATGCACACTCATTCACACAAAAAAACAGACATCCGTTGGTAATGTGTAAACTTAAATTTGCGTAAAGATTTGCTGTT

>3R:25921500..25922100

TCTTCGACTCACTCGCTCCGCATTGAGTGGGCTTTCGAGCCGTTCATGGTGCCCGGCTCGGCACGTATGCCAGTTTTGAAAAGTCCATACATACCAGACCGGGCCCAGACCAAGACCCCCAGACCTCTGCTGGGGGCATTTCCACGGAATTCCAAAACAACGTCTGCACCTGCTGCCCCATAAGTGACTAGCCGGACCACCACACCTGTGGTTAATTGAATAGGGCCACAAGCCGGAAACCAACCAACTACCAACTAGCAATGAGCAGTTCGCTTTATGGATGCAGTATCTTGAAGATATTGATTCTGCTCGGAATTGAAATACTTGTATGCTACTATGTTGACCCCTGCTGTGCCTATCTATTTGTCTGCAAATCCAGTAGAAGCGACGTGAGTGAAACGGCGTCGCCCCTTTCGATTTCACGAATTCGTCGTCTATTGAGCTCGTTTCGCAAATTGTTGGCAGAATTACGTGGCAGCTGAGCGAGACAGCAACAACATTGGGCTGCAACCACCGGCGGAACTTATGAAATAATTCTCCAGATGCCAAGCATATACAAGTTGGGGCATCAGGGGATTTGGGGGTTAGCTGATATGCACA

>3R:27050000..27050500

GAGAAGTCGTGAATGTCTGGGGTTATCAACGGCAAAATAATAATAACAATAACGAAGGCGGAGCAGAAATATAAAAAAAAACCCGAACCGGGTGAACTGATTTGACCGTTGACAAATGACGCCGTGTTCTCGATAGGATGAGGGGCGTGGCACATCAATTTCATCAGCTAGAATACGTGTATATAATTGCAGTTTTAAGTGACGAGTGTCCAAGGCAATTGATACTTGCTGAAATGTTGCCCAAAGTACGTGGAACTATTACGCACATATGGTGTGTCTGTATGTCCGCATGTTCGCATGTCCATATGTGTCATCCATCGATTGCCGGCACGTAGACATCATCGCAATCACTCCCGCAAATGTTTTTAATCGCCTTTCGGCTGATAAGCGAGCAGAGTGGCGGGGCGCGGGAGTCTTCAGATGAAAGCATATGTTTCGCATAATAGCTAATTTGCGGCATATGTTATACGTTTGCCTGCGAGTACAAATCGAAGTAAGTA

>X:9767000..9767500

CCGCACACCCGCTGAAATTTCCCGAAAATGTGACCGAATAATGGTCTTGCCAGAGCAACTGAGTGTTATTATTATAGCGCTGAGTCAGAAGTGTTCCTCGACTCATCGTTATCTCATTGACGCCGGAGTTGTCGGGTCTAGTGCTCTGTGTATAGTATCTTGTATCTTGTATCTTGTATCTGCGTTATATCTGCAGAAACCCATAGAGCCCAGTATTTCATCATACTTTTCGTGATCGACGACACGTGATGTGACGTAGTTTCGATTTCATGACGAAGCGAAAGGGTTAATGTCCATTGGGATCGTACCATCTGGGGATTTCTACATGTGAAACATACTTATGCAATAGGCCATAATAGTTTTAGCAAAGCCTTGAGCCCTTGAGAAAGCTTTCTTTTCAGAACAACTACTTTTCTTTACGATAATAATATTTGTTCATAATGAATTATATATTAAATTAATTATTGATTTTCTGCCTAATTACTTATACATTTAAGAAA

>X:16559200..16559700

GTGTGCTTTTGCCCTTGCAGGTGGAATCCGTGCAGTGGACATTCGACAAGACGACTCCCGATGACATCAACGGTGAGCTGAGACACCTGGCCGAGTTCCTGGCCAACAAGCAGTTCGACCTGGTCATCAACCTCCCGATGAGCGGCGGGGGTGCCAGGCGGTGAGTACGATTCGATGGTGACTCTTTTTAATGGCCCCCAAATATATATGCCACTAAGTTGGTGATCCATGGAACTTCAGGAAGAAGTCTGCAAGTGCGAATAGTATTTGTTTTCTAATTGTGTTTACCTATAAAACATATATAACATATTTCTTGTAGCGGATATGCATATTTATATAGCTAACCTACTTTGATAAAAGTTCTGTTTTCAGGGGTATTAACGACTTAATGATTCAATATTCAAACAAATGTTAGTCACTGGAATTTTTGGGCCTTGAACACAGTTTGCACAACAATTTGGCATATAATTTAAGTTATTAAGTTATAAAAAGGGCTTGTC

>X:17071000..17071300

TTGCCTAAAGTTTTACAGTCACTGGCCGAACAATGGGAATTTGTAAATGAATGAATGATGGAGCCAAAGAAAGGCAAAGATCTAATTGCTCAAGAACGGAAACTTACGACTTGATTGATGACTCACCTGATTGAGTGGTGAGCGGACTAAGATAAACTTGTGCGAAATGAATTAATTAAGTGCAGCCAGAGCCTGATGAGCTTCAAATCACACAAAACTGACAACTCACTCTTTCTCTTCATTTGGTCAGATGCATAATTATGTCGCTAATGGTGCCAACAAATCGGGTACAGTGAACAC

>X:17388000..17388500

TTCTCACAACGGCTGTAAAAAGTGCCACCCAAGTGGAAAACAAAAGGCGAGCAGCACAAAGGCGTTGAGAGCCAATTAATGGGAACGCCTCAATAACCGACTGTCGGAATCAGTAATTGACAAAACGACTGATTGGCCGACTGACTAACTGACTAACTGACTAACTGACTGACTGAAGGACTGACTAAGTGACTGCGAACGAGTGCGTCAATTGGCATCATCGTTAAAATGATAGAGAGCGGCAATTCTATCAGCGCGTGCAATTGTCAATTAAAGTGCGAGCTGCAAGTAATTAATGGCAAGCGAACAGAGTCCAAACACTATCAAACTCGAAAATTCATATTCACACTGGCAACTCGAAAAGTACACCGAGAAAAATGCACAAAATTGGTATCCAACGTAAAATATACACACAGAAAAGCTAGAAAAAAAAAACAAACCAAACAAAAAATACCCTGCAAATAAATGAATTAAGTTTTGAGTCTATTAGGTTCTTTATA
