# Supplementary material for: Genome-wide identification of hypoxia-induced enhancer regions
Source: PeerJ. 2015 Dec 21;3:e1527. doi: 10.7717/peerj.1527 (PMC4690393; doi:10.7717/peerj.1527)
Supplement: File S5 [file peerj-03-1527-s005.zip › enhancer_analysis_pipeline/count_data_by_100_bp_bin/READ ME count_data_by_100_bp_bin.docx]

Note: “randomer_genomic_weighted_match_list” file output from the “bowtie to matchlist pipeline” and the “bad_matches” file from the “dixon_outlier_test” pipeline must be present in the folder. The folder must also contain the output randomer lists from each experimental replicate from the “experimental_read_miner” module. This pipeline also requires use of six processors so if this is not possible the script must be changed.

Run ***count_data_by_100_bp_bin_generator_shell.pl*** in folder with files named as quoted in bold

“**N1_reads**” : untreated1 experimental randomers

“**N2_reads**” : untreated2 experimental randomers

“**N3_reads**” : untreated3 experimental randomers

“**H1_reads”** : treated1 experimental randomers

“**H2_reads**” : treated2 experimental randomers

“**H3_reads**” : treated3 experimental randomers

“**bad_matches**” : outlying randomer-100bp bin matches

“**randomer_genomic_weighted_match_list**”: match list linking randomers

to the genomic regions whose activity they report

This shell script runs ***data_hundo_allocator_from_weighted_list_hundo_stranded_corrected.pl***

to calculate activity of each 100bp bin in each condition based on experimental randomer counts and the values in the randomer_genomic_weighted_match_list. Matches from the outlier list “bad_matches” are ignored. The shell script then runs ***chromosome_displayer.pl*** and ***prepare_for_DESeq_chromosomes.pl***  to consolidate the data by replicate and chromosome into a count data matrix.

The output from this shell script is “counts_table_for_DESeq” which must be placed in the “DESeq” folder in order to perform a negative binomial test.
