# Supplementary material for: Genome-wide identification of hypoxia-induced enhancer regions
Source: PeerJ. 2015 Dec 21;3:e1527. doi: 10.7717/peerj.1527 (PMC4690393; doi:10.7717/peerj.1527)
Supplement: File S5 [file peerj-03-1527-s005.zip › enhancer_analysis_pipeline/creation_of_randomer_genomic_match_list/bowtie_to_match_list/READ ME bowtie to randomer genomic match list.docx]

This module uses the output from the bowtie2 alignment to create a file consisting of a list of tab separated lines(rows) where the first column is a randomer followed by the genomic locations it maps to and their respective weights(weight equals number of a randomer-genomic linkage divided by total number of that randomer in the paired-end match sequencing). This weighted list is used to link randomer tags to the test sequences whose activity they report. The shell script ***bowtie_to_matchlist_shell.pl*** accepts the bowtie2 output file resulting from aligning the fasta file from the “Illumina_to_bowtie” module and a user specified maximum number of repeats integer as arguments. The shell script creates a matching file called “randomer_genomic_weighted_match_list.” This file must be placed in the “dixon_outlier_test” folder as well as the “count_data_by_100_bp_bin” folder by the user.

The subscripts of the shell script are described below:

1.) Run ***randomer_genomic_bowtie2_alignment_repeat_non_repeat_separater.pl***  to separate repeats from non-repeat alignments.

2.) On the repeats from step one run ***lesser_alignments_filter.pl*** to remove repeats with lower alignment scores than the optimal repeats. Then run ***repeat_loc_liner.pl***  to collapse all alignments into a single line containing the barcode followed by all repeat locations. Then ***run repeat_region_number_limiter.pl*** to list all repeats which are inside an acceptable threshold and ***repeat_region_number_limiter_bad_repeats_printer.pl*** to print to track those which are being thrown out. Then run ***rep_sorter.pl*** to sort repeats by genomic position so that they can be accessed by a single randomer<->genomic regions pair. Then run ***repeat_singleton_separater.pl*** to remove and list those alignments that only had one optimal repeat as they will be treated like non repeats.

3.) On the non-repeats output from step one run ***non_repeats_single_printer.pl***  to reduce each alignment to the randomer and either the genomic position or the word “unaligned” if no alignment was found.

4.) ***Concatenate*** output from step three(uniquely aligned and unaligned) with highly repetitive alignments and singletons from step two. We now have one file containing “proper repeats” and one file containing unique alignments and bad alignments.

5.) Run ***randomer_genomic_weighted_list_maker_bowtie2_all.pl*** to create the final randomer_genomic_weighted_match_list.
