# Supplementary material for: Genome-wide identification of hypoxia-induced enhancer regions
Source: PeerJ. 2015 Dec 21;3:e1527. doi: 10.7717/peerj.1527 (PMC4690393; doi:10.7717/peerj.1527)
Supplement: File S5 [file peerj-03-1527-s005.zip › enhancer_analysis_pipeline/creation_of_randomer_genomic_match_list/Illumina_to_bowtie/READ ME Illumina to Bowtie.docx]

In order for the enhancer screen to function randomer tags must be linked to the potential enhancers they report for. This is done by paired-end sequencing the library. This module is used to extract and quality filter paired-end sequences matching randomer tags to their respective genomic locations and then prepare them for alignment using Bowtie2. Use shell script ***illumina_to_bowtie_shell.pl*** with arguments specified in the script header to go directly from raw data fastq files to the final fasta file. The user then aligns this fasta file(called randomers_genomics.fa) to the Drosophila genome using bowtie2 with the “find all alignments” “-a” parameter.

Information on the component scripts is given below if you wish to modify them.

1.) Run ***shell_concatenate.pl*** with arguments “$name_R1”, which is a common string in all first end read names used to grab all files from the lane, and an argument name for the output file. The script then looks through all paired end sequences for a 15bp signature in Read2 which is an invariable core promoter sequence that is present in all genomic-randomer matching sequence pairs.

2.) Run ***match_library_pe_QF.pl*** on shell_concatenate.pl output to filter read pairs by PHRED score. Other arguments are max percent chance of a single error in the barcode stretch of read 2, max percent chance of a single error in the genomic read 1, and minimum length of genomic read. The script writes the reads passing all filters to an output file specified as an argument.

3.) Run randomer_***genomic_fasta_maker_from_fastq.pl*** on filter-passing output. This creates a fasta file with the 20bp randomer as the name and the first 50bp of the genomic sequence as the sequence. This way the randomer and genomic fragment stay associated. This file is then aligned to the Drosophila genome using bowtie2 with the find all alignments “-a” parameter.
