# Supplementary material for: Genome-wide identification of hypoxia-induced enhancer regions
Source: PeerJ. 2015 Dec 21;3:e1527. doi: 10.7717/peerj.1527 (PMC4690393; doi:10.7717/peerj.1527)
Supplement: File S5 [file peerj-03-1527-s005.zip › enhancer_analysis_pipeline/creation_of_randomer_genomic_match_list/READ ME creation_of_randomer_genomic_match_list.docx]

First run the Illumina_to_bowtie module on the paired-end fastq files matching randomer tags to test sequences. This will create a fasta file that you then align to the Drosophila genome using bowtie2 with the “-a” all alignments parameter.

Second, run the bowtie_to_match_list module on the bowtie2 output mentioned above in order to create the randomer_genomic_weighted_match_list file. This file needs to then be placed by the user into the “dixon_outlier_test” and “count_data_by_100_bp_bin” folders.
