# Supplementary material for: Genome-wide identification of hypoxia-induced enhancer regions
Source: PeerJ. 2015 Dec 21;3:e1527. doi: 10.7717/peerj.1527 (PMC4690393; doi:10.7717/peerj.1527)
Supplement: File S5 [file peerj-03-1527-s005.zip › enhancer_analysis_pipeline/DESeq/READ ME DESeq.docx]

This is a simple R script which scans the “counts_table_for_DESeq” output from the “count_data_by_100_bp_bin” module for differential enhancer activity between the two conditions by performing a negative binomial test. Place the “counts_table_for_DESeq” in this folder and run ***DESeq_negative_binomial_shell_chromosomes_filter_args.R*** with argument 1 being theta, the proportion of low count bins to not include. For example if you don’t want to use the bottom thirty percent of loci by total count use a theta of 0.3. Argument 2 is the name chosen by the user of the output file with p-values, adjusted p-values, fold change, mean etc.

Be sure that Bioconductor and DESeq are installed in your R build.
