# Supplementary material for: Genome-wide identification of hypoxia-induced enhancer regions
Source: PeerJ. 2015 Dec 21;3:e1527. doi: 10.7717/peerj.1527 (PMC4690393; doi:10.7717/peerj.1527)
Supplement: File S5 [file peerj-03-1527-s005.zip › enhancer_analysis_pipeline/dixon_outlier_test/READ ME dixon outlier test.docx]

1.) Run ***experimental_randomer_counter.pl***  with randomer_genomic_weighted_match_list and each of the six replicate randomer files (output randomer lists from "experimental_read_miner" pipeline e.g. N1_reads, N2_reads etc.) as arguments to generate a list of randomers with their genomic mappings as well as their counts in each experimental condition. This creates an output file, called "randomer_genomic_weighted_match_list_with_replicate_counts", that is then used for the Dixon Q test for outliers.

2.) Run ***dixon_outlier_test.pl***. This script looks through the file "randomer_genomic_weighted_match_list_with_replicate_counts" to find randomers with outlying activity with respect to the other randomers that map to a given 100bp bin using the Dixon Q test and prints the outlying 100bp bin-randomer pairs to a file called “bad_matches”. These outliers are likely bleed through from randomers that are also linked to other genomic regions that have legitimate activity. Removing them reduces the noise of the system and makes true hits easier to identify. “bad_matches” must then be moved by the user to the “count_data_by_100_bp_bin” folder.
