# Supplementary material for: Genome-wide identification of hypoxia-induced enhancer regions
Source: PeerJ. 2015 Dec 21;3:e1527. doi: 10.7717/peerj.1527 (PMC4690393; doi:10.7717/peerj.1527)
Supplement: File S5 [file peerj-03-1527-s005.zip › enhancer_analysis_pipeline/experimental_read_miner/READ ME experimental_read_miner.docx]

1.) Run ***experimental_read_miner_se.pl***  to generate a list of randomer tags from single end Illumina fastq files. These tags represent the counts of each randomer in experimental RNA and are used to infer enhancer activity. Arguments are (1) the base name of the fastq files, i.e. a shared string all files contains in order to open them iteratively, (2) an “experimental barcode” which is a unique and consistent sequence at the beginning of the read added to each replicate during PCR amplification so that different replicates can be sequenced on the same lane, and (3) an output file which contains each randomer followed by a carriage return. The pipeline is designed for three replicates from two conditions. In order for the randomer output files to be read by downstream shell scripts name them as such:

untreated1: “N1_reads”

untreated2: “N2_reads”

untreated3: “N3_reads”

treated1: “H1_reads”

treated2: “H2_reads”

treated3: “H3_reads”

These files must then be copied to downstream module folders “count_data_by_100_bp_bin” and “dixon_outlier_test”
