# Supplementary material for: Genome-wide identification of hypoxia-induced enhancer regions
Source: PeerJ. 2015 Dec 21;3:e1527. doi: 10.7717/peerj.1527 (PMC4690393; doi:10.7717/peerj.1527)
Supplement: File S5 [file peerj-03-1527-s005.zip › enhancer_analysis_pipeline/method pictorial.pdf]

A nucleic acid barcoding method to assay genome-wide enhancer activity

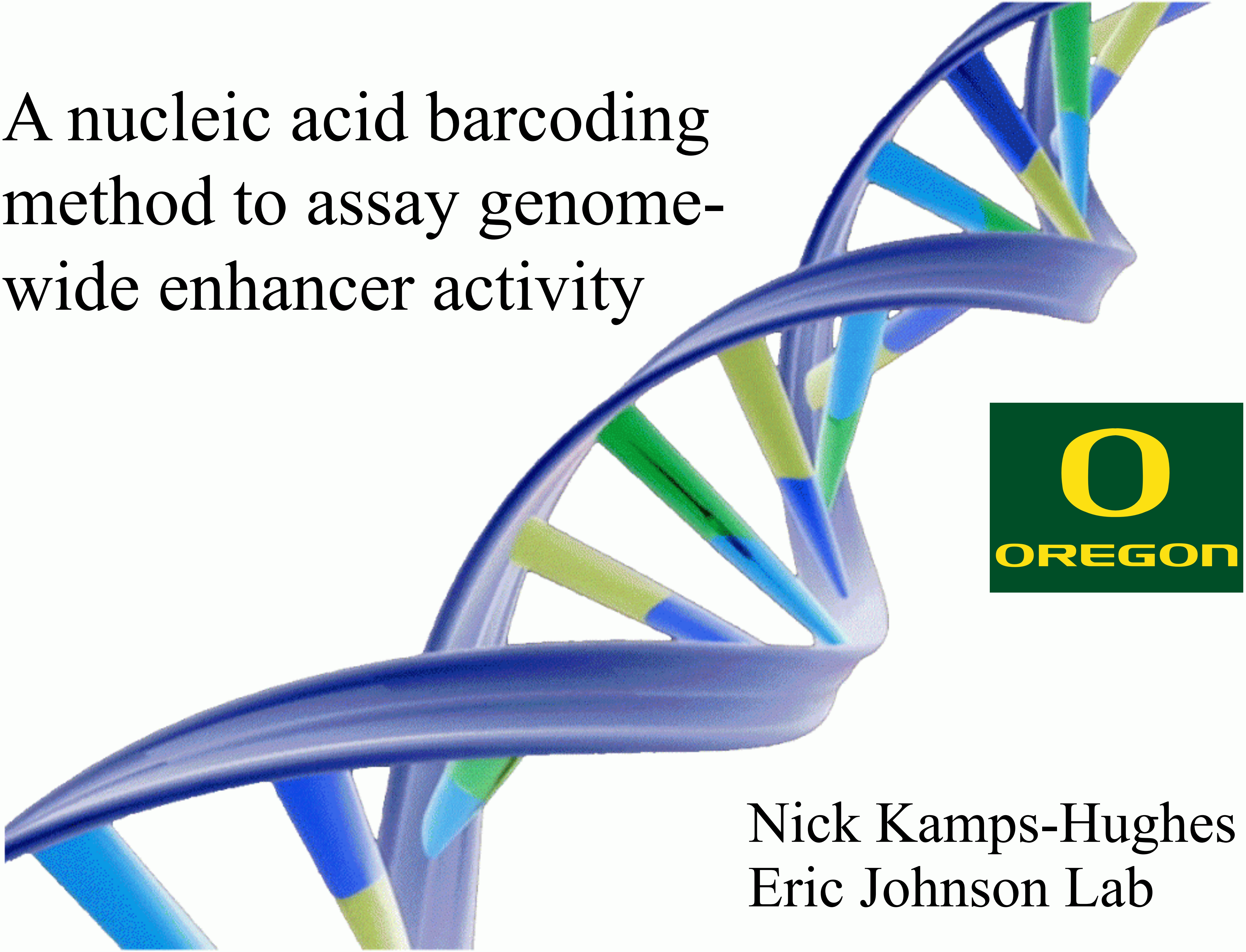

Nick Kamps-Hughes
Eric Johnson Lab

Enhancer Library Synthesis

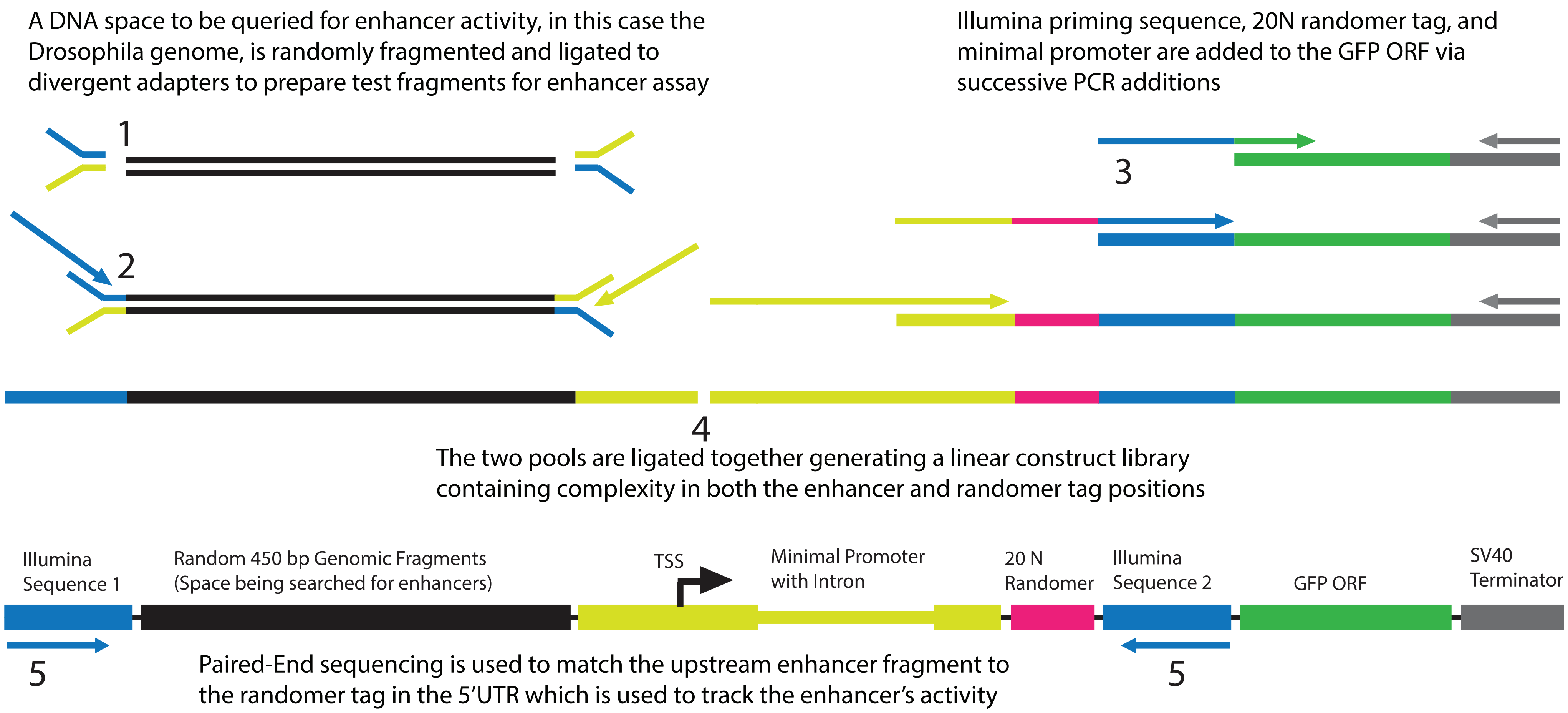

Enhancer Library Assay

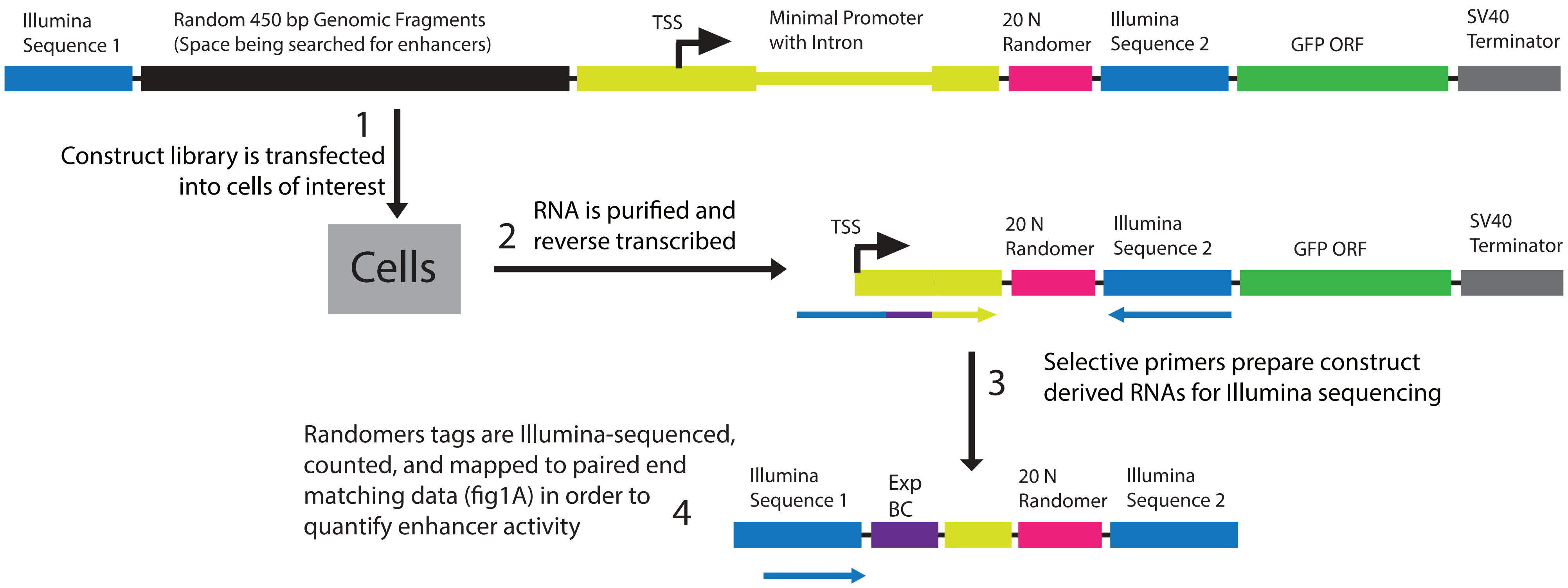

Bioinformatics Pipeline

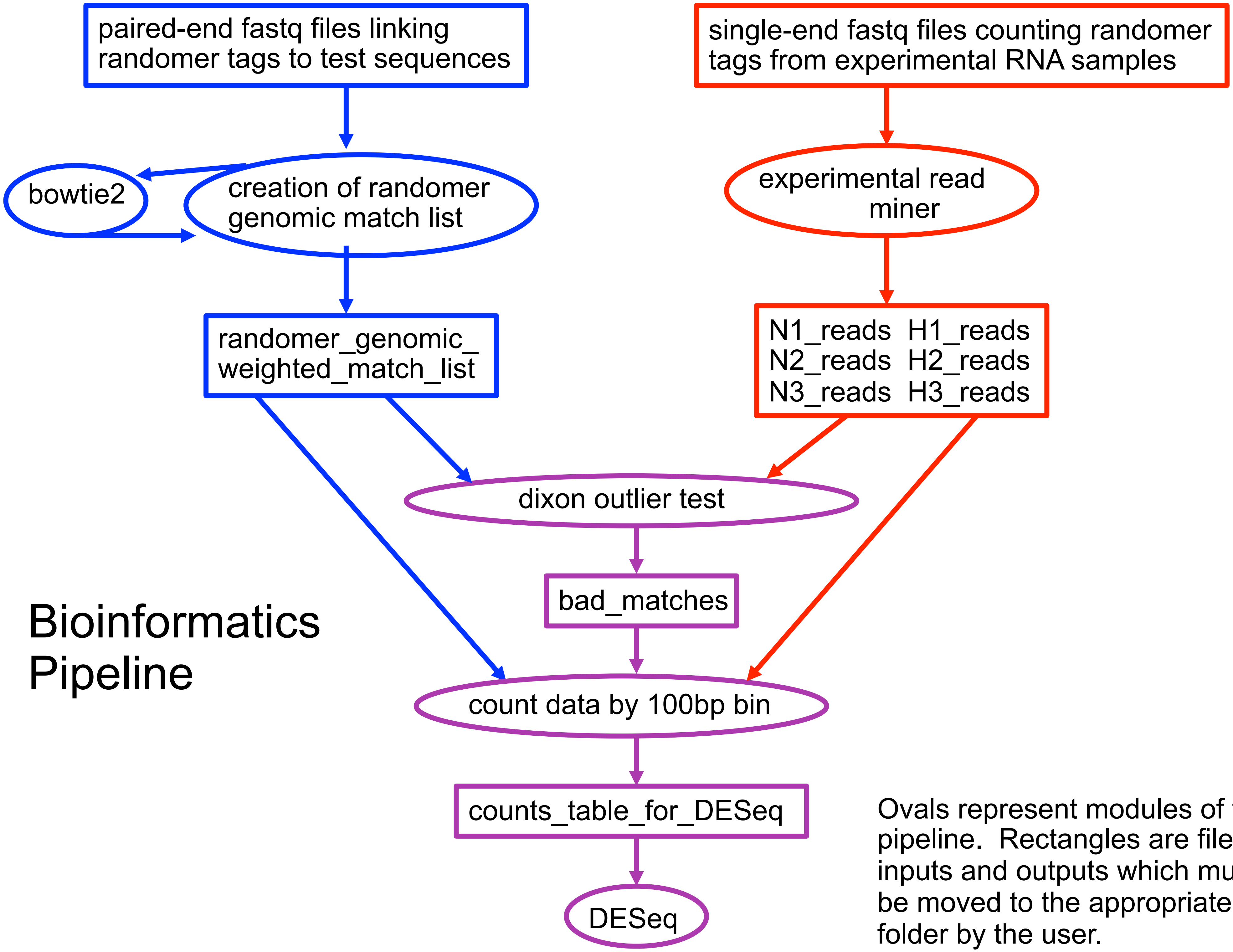

Ovals represent modules of the pipeline. Rectangles are file inputs and outputs which must be moved to the appropriate folder by the user.
