# Supplementary material for: Genome-wide identification of hypoxia-induced enhancer regions
Source: PeerJ. 2015 Dec 21;3:e1527. doi: 10.7717/peerj.1527 (PMC4690393; doi:10.7717/peerj.1527)
Supplement: File S5 [file peerj-03-1527-s005.zip › enhancer_analysis_pipeline/READ ME Enhancer Pipeline.docx]

This document describes the general components of the enhancer analysis pipeline. Each module is described in more detail within its respective folder. Also, see the “method pictorial” provided to help understand the molecular protocol and for a schematic of the bioinformatics flow. Note that the analysis pipeline is set up for analyzing differential enhancer activity from the specific construct library used in the experiments described in this paper in a two condition, three replicate format. Changes to construct sequence features, input genomic DNA library, or other experimental design components will necessitate changing components of the computational pipeline accordingly.

**Module 1: creation_of_randomer_genomic_match_list**

The experimental method involves synthesizing a library wherein millions of unique potential enhancer regions(test sequences) are linked to millions of unique randomer “tags” in the 5’ UTR. The tags are sequenced from experimental RNA in order to quantify the enhancer activity of the test sequence. In order for this to be possible, test sequences and randomer tags are linked through paired-end sequencing. This module of the pipeline turns fastq files containing paired-end reads linking randomer tags with test sequences into a table where each randomer is matched to its respective genomic position(s).

**Module 2: experimental_read_miner**

The enhancer library is transfected into cells and mRNA is then extracted from these cells and an amplicon containing the randomer tags is sequenced in order to count tags and infer enhancer activity of test fragments. This module contains a script for extracting these randomer tags from single-end fastq files.

**Module 3: dixon_outlier_test**

Many randomers correspond to multiple genomic regions due to bottlenecks in the library synthesis. This is not insurmountable because each location of the genome is covered by many fragments. However, strong enhancers’ signal can bleed through to other loci increasing the dispersion of the data and thus increasing p-values of true hits making them more difficult to discover. This module contains scripts that filter these contaminating signals by performing a Dixon Q test on all randomers mapping to a given genomic location.

**Module 4: count_data_by_100_bp_bin**

This module contains scripts that combine the randomer-genomic match list, the outlier list and the randomer counts from each replicate to create a genome-wide count table of enhancer activity by 100bp bin.

**Module 5: DESeq**

This module contains an R script to run a negative binomial test on the enhancer activity count table in order to identify statistically significant context-specific enhancers.
